# Thyroglobulin Gene Mutation with Cold Nodule on Thyroid Scintigraphy

**DOI:** 10.1155/2012/280319

**Published:** 2012-06-03

**Authors:** Toshio Kahara, Noboru Igarashi, Akira Hishinuma, Yuko Nakanishi, Akio Uchiyama, Atsuo Miwa, Shin Ishizawa, Yutaka Yamamoto, Hirofumi Noto, Hisashi Sumiya, Kazuhide Ishikura, Rika Usuda, Hiroyuki Iida

**Affiliations:** ^1^Department of Internal Medicine, Toyama Prefectural Central Hospital, 2-2-78 Nishinagae, Toyama, Toyama 930-8550, Japan; ^2^Department of Pediatrics, Toyama Prefectural Central Hospital, 2-2-78 Nishinagae, Toyama, Toyama 930-8550, Japan; ^3^Department of Clinical Laboratory Medicine, Dokkyo Medical University, 880 Kitakobayashi, Mibu, Shimotsuga, Tochigi 321-0293, Japan; ^4^Department of Clinical Pathology, Toyama Prefectural Central Hospital, 2-2-78 Nishinagae, Toyama, Toyama 930-8550, Japan; ^5^Department of Thoracic Surgery, Toyama Prefectural Central Hospital, 2-2-78 Nishinagae, Toyama, Toyama 930-8550, Japan; ^6^Department of Radiology, Toyama Prefectural Central Hospital, 2-2-78 Nishinagae, Toyama, Toyama 930-8550, Japan

## Abstract

Thyroglobulin gene mutation is a rare cause of congenital hypothyroidism, but thyroglobulin gene mutations are thought to be associated with thyroid cancer development. A 21-year-old Japanese man treated with levothyroxine for congenital hypothyroidism had an enlarged thyroid gland with undetectable serum thyroglobulin despite elevated serum TSH level. The patient was diagnosed with thyroglobulin gene mutation, with compound heterozygosity for Gly304Cys missense mutation and Arg432X nonsense mutation. Ultrasonography showed a hypovascular large tumor in the left lobe that appeared as a cold nodule on thyroid scintigraphy. He underwent total thyroidectomy, but pathological study did not reveal findings of thyroid carcinoma, but rather a hyperplastic nodule with hemorrhage. Strong cytoplasmic thyroglobulin immunostaining was observed, but sodium iodide symporter immunostaining was hardly detected in the hyperplastic nodule. The clinical characteristics of patients with thyroglobulin gene mutations are diverse, and some patients are diagnosed by chance on examination of goiter in adults. The presence of thyroid tumors that appear as cold nodules on thyroid scintigraphy should consider the potential for thyroid carcinoma, if the patient has relatively low serum thyroglobulin concentration in relation to the degree of TSH without thyroglobulin autoantibody.

## 1. Introduction

Thyroglobulin (Tg) is a large glycoprotein synthesized by the thyroid gland and functions as a matrix for thyroid hormone synthesis. Correctly folded Tg homodimers are secreted into the follicular lumen where coupling between either two diiodotyrosine residues or between a diiodotyrosine and a monoiodotyrosine residue results in the formation of T4 or T3. Dyshormonogenesis due to Tg gene mutation is a rare cause of congenital hypothyroidism, and analysis of patients in Kumamoto Prefecture in Japan showed that the incidence of Tg gene mutations is one in 67,000 individuals [1]. Patients with Tg synthesis defects present with congenital goiter, hypothyroidism or euthyroidism, high radioactive iodine uptake, normal organification of iodide, elevated serum TSH with simultaneous low serum T4 levels, and low serum Tg concentration in relation to the degree of TSH stimulation [2]. Hishinuma et al. report that the Tg gene mutations are associated with thyroid cancer development, and 63.6% of the patients with Tg gene mutation who undergo surgery have thyroid carcinomas [3]. We experienced a case of Tg gene mutation with cold nodule on thyroid scintigraphy, who had compound heterozygosity of Tg gene mutation for Gly304Cys/Arg432X. On surgical examination, a hyperplastic nodule in a dyshormonogenic goiter was found, and we consider the findings of immunohistochemical study. 

## 2. Case Report 

**Figure 1:**
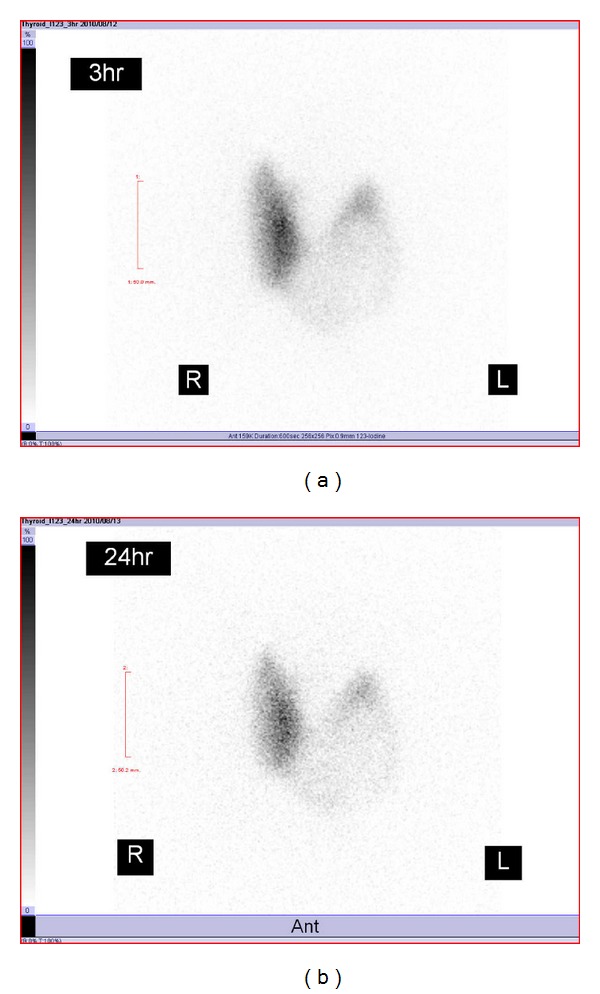
123-I scintigraphy. Radioactive iodine uptake was increased (3 hours; 59.2%, 24 hours; 74.9%), and the left thyroid tumor appeared as a cold nodule.

**Figure 2:**
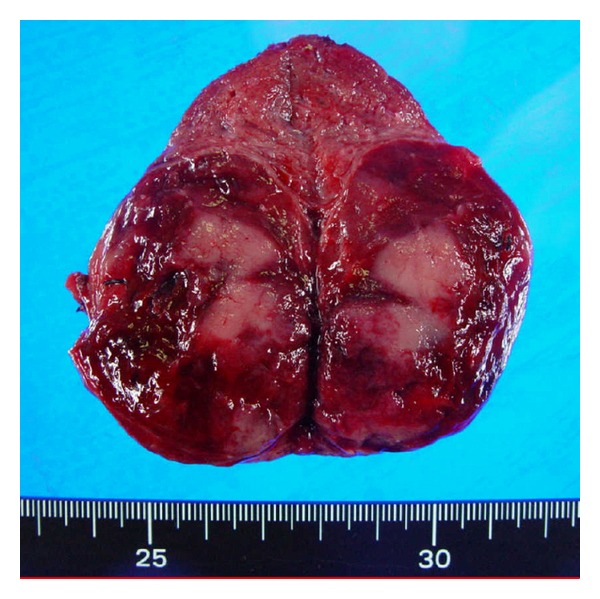
Surgical specimen. The left thyroid tumor showed a solid nodule with extensive hemorrhage.

**Figure 3:**
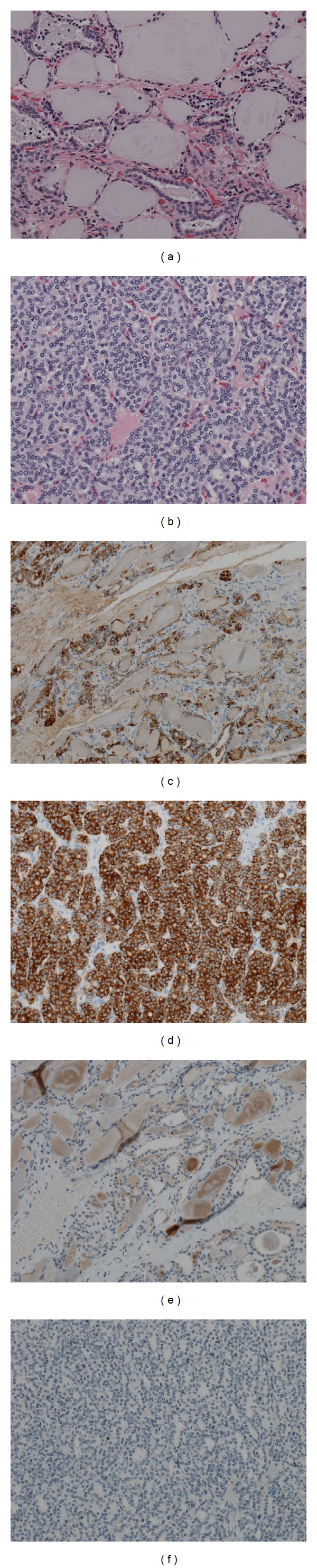
Pathological findings (original magnification ×400). In the surrounding thyroid tissue, the color tone of the follicle colloid was a light bluish color ((a), hematoxylin and eosin). The thyroid tumor consisted of mainly compact collections of small follicles that lacked colloid ((b), hematoxylin and eosin). Strong cytoplasmic Tg immunostaining was observed in both the surrounding thyroid tissue (c) and the hyperplastic nodule (d), although its appearance in the follicle lumen was deficient. NIS immunostaining was observed in the surrounding thyroid tissue (e). On the other hand, NIS immunostaining was hardly detected in the hyperplastic nodule (f).

The patient was born by a spontaneous vaginal delivery at 39 weeks of gestation, and his birth weight was 2,900 grams. There were no complications during the pregnancy. The patient had a goiter and he was diagnosed with cretinism based on levels of TSH 57.2 *μ*U/mL and T4 2.9 *μ*g/dL on day 10. His parents had no past history of thyroid disease, and they do not have a consanguineous marriage. He had been treated with levothyroxine, and he grew up without developmental disturbance or intellectual impairment. However, his goiter was gradually aggravated, and a detailed examination was performed at 21-year-old. The large goiter was not tender and was elastic soft. The left lobe was enlarged remarkably compared to the right lobe. He was taking 100 *μ*g/day of levothyroxine, and blood analysis indicated elevated TSH of 8.43 *μ*U/mL, free T3 of 3.4 pg/mL, and free T4 of 0.9 ng/dL. Thyroid autoantibodies against Tg, thyroid peroxidase, and TSH receptor were negative. The serum Tg concentration was undetectable, suggesting that hypothyroidism could be related to defective Tg synthesis. Informed consent was obtained, and genomic DNA was isolated from peripheral blood leukocytes. DNA sequencing identified the presence of compound heterozygosity for Gly304Cys missense mutation and Arg432X nonsense mutation in the Tg gene. Thyroid ultrasonographic study showed a solid tumor more than 5 cm in the left lobe. The right lobe was hypervascularized, but the left thyroid tumor was hypovascularized. His radioactive iodine uptake was increased, but the left thyroid tumor showed a cold image (Figure 1). Fine-needle aspiration cytology in the left thyroid tumor did not reveal findings of malignancy. As the incidence rate of thyroid cancer in patients with Tg gene mutations is high [2], this patient underwent total thyroidectomy. On surgical examination, the thyroid tumor was a solid nodule with extensive hemorrhage (Figure 2). On light microscopic examination with hematoxylin and eosin staining, the color tone of the follicle colloid in the surrounding thyroid tissue of the tumor was not normal acidophil but rather a light bluish color, similar to the findings of dyshormonogenic goiter (Figure 3(a)). On the other hand, the left thyroid tumor revealed no findings of destructive thyroiditis, and the tumor consisted of mainly compact collections of small follicles without colloid (Figure 3(b)). The nuclear findings did not suggest papillary carcinoma. No findings of vascular invasion or extracapsular invasion such as follicular carcinoma were found. The thyroid tumor was thought to be a hyperplastic nodule because there was no consecutive capsular film. Strong cytoplasmic Tg immunostaining was observed in both the surrounding thyroid tissue (Figure 3(c)) and the hyperplastic nodule (Figure 3(d)), although its appearance in the follicle lumen was deficient. Sodium iodide symporter (NIS) immunostaining was observed in the surrounding thyroid tissue (Figure 3(e)). On the other hand, NIS immunostaining was hardly detected in the hyperplastic nodule (Figure 3(f)). Consequently, this finding may be related to defects of the iodine-trapping ability in the hyperplastic nodule. Ki-67 activity in the hyperplastic nodule indicated minimal cell proliferation. The activating point mutation V600E in exon 15 of the BRAF gene was not recognized in the hyperplastic nodule. 

## 3. Discussion 

There is an inverse correlation between accumulation of follicular Tg and radioactive iodine uptake, and follicular Tg accumulation is thought to suppress NIS gene expression [4]. Because patients with Tg gene mutation present with decreased follicular Tg accumulation and elevated serum TSH, NIS gene expression is thought to be increased and thyroid scintigraphy shows a diffuse goiter with increased radioactive iodine uptake. On the other hand, thyroid cancer appears as a cold nodule on thyroid scintigraphy, and the number of NIS-positive cells in thyroid carcinoma is significantly lower than in normal thyroid tissue [5]. 

Thyroid tumor in this case with Tg gene mutation was not thyroid cancer but a hyperplastic nodule, and the tumor appeared as a cold nodule on thyroid scintigraphy. On immunohistochemical study, strong cytoplasmic Tg immunostaining was observed, but NIS immunostaining was hardly detected in the hyperplastic nodule. At this point, the hyperplastic nodule was different from the surrounding tissue in character. 

Because atypical cellular findings are often found in hyperplastic nodular legions, it is sometimes difficult to differentiate malignant transformation from benign hyperplasia. The activating point mutation V600E in exon 15 of the BRAF gene was not recognized in the hyperplastic nodule of this case, but the prolonged TSH stimulation might promote malignant transformation and development of thyroid cancer in dyshormonogenic goiter [6]. 

## 4. Conclusion

Tg gene mutations are thought to be associated with thyroid cancer development. Clinical characteristics of patients with Tg gene mutations are diverse, ranging from congenitally hypothyroid patients detected in fetal life to adult goitrous patients with normal thyroid function. Some patients are overlooked in initiation of neonatal screening and are diagnosed by chance on examination of goiter in adults. The presence of thyroid tumors with cold nodules on thyroid scintigraphy should consider the potential for thyroid carcinoma, if the patient has relatively low serum Tg concentration in relation to the degree of TSH without Tg autoantibody.
